# Effect of *Melia azedarach* (Sapindales: Meliaceae) fruit extracts on Citrus Leafminer *Phyllocnistis citrella* (Lepidoptera: Gracillariidae)

**DOI:** 10.1186/2193-1801-2-144

**Published:** 2013-04-05

**Authors:** Maher M Mckenna, Efat M Abou-Fakhr Hammad, Mohamad T Farran

**Affiliations:** 1 Department of Plant Protection, Faculty of Agriculture and Veterinary Sciences, Lebanese University, Beirut, Lebanon; 2 Department of Agricultural Sciences, Faculty of Agricultural and Food Sciences, American University of Beirut, Beirut, Lebanon; 3 Department of Animal & Veterinary Sciences, Faculty of Agricultural and Food Sciences, American University of Beirut, Beirut, Lebanon

**Keywords:** Lemon, Leafminer, Botanical, Chinaberry, Biorational, Larvae

## Abstract

*Melia azedarach* L. extracts were studied in comparison with selected biorational insecticides against the citrus leafminer *Phyllocnistis citrella* Stainton under field conditions. *Citrus limon* (L.) Burm. F. trees were exposed to: *Melia* extracts of green and mature fruits, Neem oil (30% a.i.), abamectin (1.8% a.i.) and control. Two sprays of each treatment (except for *Melia* mature fruit extract) were executed at 10-d intervals. The live number of the 1^st^ and later (2^nd^ & 3^rd^) larval instars per leaf were recorded at initial sampling date and at 10-d intervals after each spray application. Results indicated that there were significant differences in the number of live larval instars among treatments. *Melia* extracts and the two biorationals, neem oil and abamectin, decreased the larvae population significantly to lower numbers than that of the control at 10 days after each spray application. However, the decrease caused by neem oil and abamectin was significantly higher than that of *Melia* extracts. Thus, these extracts might be considered as potential alternative with other biorational control methods in management of the leafminer. Further research including bioassays is needed to determine the factors responsible for reducing larvae population and whether these *Melia* extracts can be utilized in future citrus IPM programs as a tool for citrus leafminer management.

## Introduction

The citrus leafminer (CLM) *Phyllocnistis citrella* Stainton (Lepidoptera: Gracillariidae: Phyllocnistinae) is one of the major constraints to citrus production in the world. Effective chemical control of the CLM is limited as the feeding larvae are protected by the leaf cuticle mainly in mines of immature foliage and the pupal stage is also protected by the rolled leaf margins (Jones 2001; Beattie 2004). Several pressures as pesticide resistance has accelerated the search for safe environmentally, toxicologically, selective and efficacious pesticides (Weinzierl and Henn 1991; Dias et al. 2005; Amiri-Besheli 2008, 2009). Biopesticides, including botanicals, can offer a safe and effective alternative to conventional insecticides controlling major insect pests. Botanicals are a promising source of pest control compounds. The pool of plants possessing insecticidal substances is enormous (FAO 1992; Jacobson 1989). These have generated remarkable interest in recent years as potential sources of natural insect control agents. Over 2000 species of plants are known to possess different degrees of insecticidal activity (Jacobson 1975). However, a few plant-based pesticides have already been utilized as plant protection products (Isman 2006). Several secondary plant products represent a large reservoir of chemical structures with biological activity against pests in form of pesticides (Grainage and Ahmed 1988; Schmutterer 1990; Lowery and Isman 1994; Miller and Uetz 1998). Throughout history, plant products have been successfully exploited as insecticides, insect repellents, insect antifeedants (Lydon and Duke 1989) or as altering insect feeding behavior, growth, development, and behavior during mating and oviposition (Yang and Tang 1988).

The Chinaberry tree, *Melia azedarach* L. (Sapindales: Meliaceae), is a deciduous tree often grown for its medicinal uses and for shade or ornament on roadsides. The non-conventional insecticidal effects of extracts or compounds isolated from Meliaceae trees include: partial reduction or complete inhibition of fecundity and egg hatchability, reduction of life span of adults, oviposition deterrence, antifeedant effects, and insect growth regulatory effects at molting (Ascher 1993). *M. azedarach* has been shown to possess many insecticidal effects against different pest species (Shin-Foon 1987; Ascher et al. 1995; Nardo et al. 1997; Valladares et al. 1997; Jazzar and Abou-Fakhr Hammad 2003; Abou-Fakhr Hammad and McAuslane 2006, 2010; Al-Akhras 2010). A limited number of studies have dealt with the use of botanicals against the citrus leafminer. Amiri-Besheli (2011) indicated that the use of biopesticides such as Tondexir® extracts from hot pepper, discouraged CLM adults from laying eggs on leaves and posed lower risk to humans and the environment than other pesticides. Pepper extracts worked better on soft bodied insects during the larval stage as the chemical was able to penetrate the leaves. Howard (1993) also found that the 2 biorationals, Avid (abamectin 0.15 EC) and Azatin (60 ppm azadirachtin) with Triton B-1956 spreader-sticker (Rhom & Haas Co., Philadelphia, PA), prevented damage by CLM when sprayed on leaves prior to oviposition by this insect; these sprays were timed just as trees were flushing.

The main strategy in plant protection scopes is to minimize the use of synthetic compounds in benefit of alternatives, such as biorational insecticides. Pesticide resistance and negative effects on non-target organisms are the main problems in pest management (FAO 1992; Franzen 1993). Natural insecticides are relatively harmless against the non-target organisms. In agricultural pest management, botanical insecticides are best suited for use in organic food production in industrialized countries but can play a much greater role in the production and postharvest protection of food in developing countries (Isman 2006). Availability of *Melia* trees allows growers to use available resources to control pests of significant economic importance. The objective of this study is to elucidate the efficacy of *M. azedarach* fruit extracts in management of the citrus leafminer larvae population under field conditions in comparison with other biopesticides.

## Materials and methods

The experiment was conducted during the summer of 2011 in an orchard of *Citrus × limon* (L.) Burm.f. Monachello lemon variety (Moussa and El Hajj 2010) located in Tyre, South Lebanon. This variety provides fruits all year round, mainly in winter and spring; the fruit is medium–small, elliptical but tapering at both ends; neck lacking; nipple small and inconspicuous with few or no seeds. Fruit color is yellow at maturity with a thin rind. The tree has nearly thornless branches with dense foliage. During the experimental period (June-July), the maximum ambient temperature ranged between 28 and 32°C whereas the minimum temperature was 20–22°C (http://freemeteo.com/default.asp?pid=15&la=19&cn=LB). Sampled leaves were examined at the Plant Protection Laboratory, Faculty of Agriculture & Veterinary Sciences, Lebanese University.

### Site description

The lemon orchard is located in Tyrdebba village, 7 kilometers from city of Tyre in South Lebanon, at an altitude of 180 meters. The orchard is adjacent to an olive orchard on the North and to an orange orchard on the South side, while roads bordered East and West sides. The orchard contained 92 lemon trees, distributed over two terraces; one terrace is 2 meters higher than the other and is separated by a steep slope. Trees were 8 years old, distributed in lines, with 3 m spacing between and within those lines. Three years prior to the experiment, trees have been treated once with Supracide® (a.i. methidathion 25%) while no other application of pesticides or chemical fertilizers has been given. In order to obtain the essential for the experiment availability of young flush, trees were sufficiently irrigated in late-April 2011.

### Preparation of M. azedarach extracts

Four Kg of *M. azedarach* green fruits and 2 Kg of *Melia* mature fruits were collected from trees at Tyrdebba village, washed and cleaned with tap water then rinsed with distilled water. Fruits were left to dry at room temperature for 2 days, and then crushed with a hammer into small pieces and ground using a grinding machine (BRAUN Multiquick 3, Hungary). The obtained material was soaked in distilled water, at a rate of 1 g per 5 ml, for 24 hours prior to spraying. The soaked material was filtered using a fine mesh (500 μm) cloth material. About twenty three liters of each of *Melia* green and mature fruits extracts were obtained after filtration.

### Experimental procedure

Four different treatments were tested: *Melia* green fruit extract, *Melia* mature fruit extract, neem oil-30% (Atlantica Agricola, Spain), abamectin 1.8% w/v – Mectin® 1.8 EC (Adonis Industrial - S.I.AD s.a.l, Lebanon). Five single-tree replicates per treatment were used, plus 5 trees treated with tap water used as control. Trees were surrounded by untreated border trees, selected so as to avoid spray drifting. All products were applied as foliar sprays to runoff (approximately 4-L), coating upper and lower leaf surfaces, using an 8-L pressure gun sprayer (Zhejiang CNG Plastic Co., China). Also, it’s worth mentioning that in windy weather, the canopy of the control trees was covered with plastic sheet to avoid spray drifting from treated trees. Neem oil and abamectin, as two positive controls, were used at the recommended dose of 0.1 μl/100 ml and 0.03 ml/100 ml, respectively. First application was made on June 16^th^ and the second on June 26^th^. In the second application, 3 treatments plus the control were tested, because there was no sufficient *Melia* mature fruit extract.

### Data collection

Three samplings were made. The first sampling was just before the first spray application, the second sampling was10 days after the first application and before the second spray, and the third sampling was10 days after the second application. In each sampling, 24 young leaves were collected from each tree; 6 from each geographical direction (North, South, East, and West), 3 from the inner and 3 from the outer part of the canopy. The latter factors were selected in order to take representative sample from each tree-replicate. Totally, 600 leaves were collected in each of the first two samplings and 480 in the third. Leaves collected from each direction and tree location were placed in separate labeled small plastic bags and kept transferred within a cooler to the Laboratory, in which they were stored at 10°C until their observation. Numbers of live 1st instar (L1) and 2nd & 3rd instar larvae (L2 & L3) per leaf were recorded. The length of the larva was used for separation of the CLM larval instars; 1^st^ instar larvae were less than 2 mm in length whereas the 2^nd^ and 3^rd^ instars were between 2 and 3 mm; in addition to their length, the latter instars were translucent and yellowish-green (Kerns et al. 2001).

### Statistical analysis

The experiment was laid out as a three-way factorial arrangement of treatments (5 × 4 × 2) for treatment, geographical direction and leaf location, respectively along with their two- and three-way interactions in a complete randomized design. As there was no significant interaction among the 3 factors, the data were pooled and analyzed as a one-way ANOVA with the treatment as the main factor. Consequently, there were 5 treatments (only 4 treatments in the third sampling) with 5 tree-replicates per treatment. The average numbers of live larvae per leaf were used in the data analysis, after ensuring their normal distribution by transforming the data using sqrt (*x* + 1) with *x* being the number of live larvae per leaf (Gomez and Gomez 1984). The analysis of data was performed using the general linear models procedure, and means were separated by Student Newman Keuls test whenever P < 0.05 was detected (SAS 1992).

## Results

None of the interaction terms among the three main factors: treatment, geographical direction and leaf location was significant. In addition, the factorial analysis showed that direction and location were not significant indicating that CLM had no preference for inner or outside flush of citrus trees nor has preference for infesting one of the directions of the tree. Consequently, data were pooled as indicated above and analyzed with the treatment being the main factor, with mean values presented in Tables 1 & 2. There were no significant differences in the initial number of live larvae of all instars at the first sampling date before applying the 1^st^ spray of each treatment. The initial counts were comparable among treatments with a range of 0.333-0.367 and 0.383-0.492 larva per leaf for the L1 and (L2 & L3) larval instars, respectively.

**Table 1:** **Effects of*Melia azedarach*fruit extracts and selected biorational insecticides on Citrus leafminer first instar larvae (L1) infesting canopy of Lemon trees under field conditions**

	Average number of live larvae (L1) per leaf*		
Treatments	Before 1st spray**	10 days after 1st spray	10 days after 2nd spray
Control	0.330 ± 0.018	0.517 ± 0.023 a	0.658 ± 0.020 a
*Melia* green fruit extract	0.330 ± 0.018	0.342 ± 0.018 b	0.400 ± 0.019 b
*Melia* mature fruit extract	0.350 ± 0.016	0.317 ± 0.019 b	---
Neem oil	0.342 ± 0.017	0.100 ± 0.011 c	0.083 ± 0.010 c
Abamectin	0.367 ± 0.016	0.058 ± 0.009 c	0.067 ± 0.010 c

Measurement values presented are mean + SEM.

* = Means in a column followed by different letters are significantly different (Student Newman Keuls test, P < 0.05).

** = Initial counts of live larvae were not significantly different at the first date of the experiment before applying the 1^st^ spray for each treatment.

**Table 2:** **Effects of*Melia azedarach*fruit extracts and selected biorational insecticides on Citrus leafminer 2**^**nd**^**& 3**^**rd**^**instar larvae (L2& L3) infesting canopy of Lemon trees under field conditions**

	Average number of live larvae (L2&L3) per leaf*		
Treatments	Before 1st spray**	10 days after 1st spray	10 days after 2nd spray
Control	0.467 ± 0.018	0.567 ± 0.022 a	0.850 ± 0.026 a
*Melia* green fruit extract	0.492 ± 0.018	0.358 ± 0.017 b	0.417 ± 0.019 b
*Melia* mature fruit extract	0.492 ± 0.017	0.350 ± 0.017 b	---
Neem oil	0.475 ± 0.018	0.108 ± 0.012 c	0.100 ± 0.011 c
Abamectin	0.383 ± 0.020	0.050 ± 0.010 c	0.033 ± 0.007 c

Measurement values presented are mean + SEM.

* = Means in a column followed by different letters are significantly different (Student Newman Keuls test, P < 0.05).

** = Initial counts of live larvae were not significantly different at the first date of the experiment before applying the 1^st^ spray for each treatment.

There were significant differences in number of L1 among treatments at 10 days after the1^st^ spray application (F = 76.77; df = 4, 24; p < 0.0001) and at 10 days after the 2^nd^ spray application (F = 104.66; df = 3, 19; p < 0.0001). Similarly, there were significant differences (F = 89.02; df = 4, 24; p < 0.0001) and (F = 58.53; df = 3, 19; p < 0.0001) in number of L2 & L3 combined among treatments at the previously mentioned dates of post spray applications, respectively.

There was no significant difference in number of live larvae per leaf between *Melia* green and mature fruit extracts, but both treatments were significantly different from the control. As shown in Table 1, the *Melia* green and mature fruit extracts have caused a significantly low population of L1 (0.342 and 0.317 live larvae per leaf, respectively) compared with the control (0.517) at 10 days after 1^st^ spray application. Also in comparison with the control, the *Melia* green fruit extract resulted in a significantly lower L1 population (0.400 Vs 0.658 live larvae per leaf) at 10 days after the 2^nd^ spray application. Similarly, the *Melia* green and mature fruit extracts have maintained a significantly low population of the L2 & L3 combined (0.358 and 0.350 live larvae per leaf, respectively) in comparison with the control (0.567) at 10 days after the1^st^ spray application (Table 2). In addition, the *Melia* green fruit extract had also maintained a low population of the L2 & L3 (0.417) as compared with 0.850 larvae per leaf for the control at 10 days after the 2^nd^ spray application.

In this study, the *Melia* extracts seem to keep the population of the CLM at a relatively constant level rather than increasing its larval numbers as in the control; the latter showed increased population of the CLM larval number throughout the experiment (Tables 1 & 2). The two commercial insecticides: Neem oil and abamectin had comparative CLM results irrespective of the spray application dates. They had, however, a significantly higher effect than the two *Melia* fruit extracts. In fact, the two insecticides significantly reduced the population of L1 to 0.100 and 0.058 and L2 & L3 (combined) to 0.108 and 0.050 larvae per leaf, respectively at 10 days after the 1^st^ spray application. It is worth mentioning that these insecticides had maintained the larval CLM population at its lowest levels throughout the experimental period (Table 1 & 2).

## Discussion

Generally, *M. azedarach* fruit extracts exert various effects on leafminer species. The population of the Agromyzid pea leafminer *Liriomyza huidobrensis* (Blanchard) was reduced after surface application of *Melia* fruit extract on swisschard and cucumber plants grown under field and greenhouse conditions, respectively (Abou-Fakhr Hammad et al. 2000a, 2000b). Similarly, *M. azedarach* immature fruit extracts resulted in fewer initiated larval mines of the vegetable leafminer *Liriomyza sativae* Blanchard on cowpea plants (Abou-Fakhr Hammad and McAuslane 2010)*.* Furthermore, *Melia* extracts applied on various plants exerted toxic and repellent effect against the nymph and adult whiteflies of *Bemisia* species, respectively (Abou-Fakhr Hammad and McAuslane 2006; Abou-Fakhr Hammad et al. 2001; Abou-Fakhr Hammad et al. 2000a, 2000b).

In the current study, both *Melia* extracts behaved similarly as they yielded comparable results 10 days after the first spray application but unfortunately, the *Melia* mature fruit extract could not be tested thereafter. Extracts of different ages of *Melia* fruits were comparable in their effect against the CLM after the 1^st^ spray application similar to extracts of different ages of *Melia* (Abou-Fakhr Hammad et al. fruits and leaves tested against *B. tabaci*^2001^). The significantly lower number of CLM live larvae in both fruit extract treatments in comparison with the control (Tables 1 & 2), could have been caused by the ingestion of the *Melia* components present in the leaf mines. The fact that the number of larvae remained relatively constant after the second spraying with the green *Melia* extract may indicate a potential toxic or antifeedant effect of this extract. In comparison with the two biorational treatments, however, both *Melia* extracts were less efficient after 10 days of the first application and this efficiency did not improve even after the second spray of the *Melia* green fruit extract. This could be an indication of either low concentrations of the extracts reaching the mines or the result of a weakening effect of the active ingredients of the *Melia* extracts in the plant tissues with time. A weakening effect of azadirachtin (of initially 0.17 g active ingredient/cm trunk diameter) against the litchi stink bug pest, *Tessaratoma papillosa* Drury, in the plant tissues after about 2–3 weeks was reported by Schulte et al. (2006). Furthermore, the persistence of azadirachtin was prolonged in systemic applications compared with its residual life (4–8 days) when foliar sprayed (applied in the current study) under field conditions (Ascher 1993). In addition, the *Melia* extracts used in this study may have lacked the rapid penetration and translaminar action through the leaf tissues; characteristics of neem oil and abamectin, respectively (Mujica et al. 2000).

Different commercial insecticides including botanicals and other biorationals have been tested for the control of CLM. These products differ in their formulation and concentration of the active ingredients. Azadirachtin (as Margson-O®; 25% a.i.), abamectin and mineral oil had a comparable effect against CLM (L1, L2 & L3) on navel oranges under field conditions after 2^nd^ spray application (Abou-Fakhr Hammad and Antar 2003). Howard (1993) reported similar CLM results on Lime seedlings treated with the two biorationals, Avid® (abamectin 0.15 EC) and Azatin® (60 ppm azadirachtin) with Triton, at the flushing period. Plants sprayed with abamectin, however, remained completely free of leafminer damage, whereas 33 and 50% of azadirachtin treated leaves showed incipient mines 3 and 4 weeks post spraying, respectively; the incipient mines did not progress when observed 5 weeks post spraying with no leaf curling detected. These results indicate that botanical products including *Melia* plant extracts need more time to reveal their insecticidal effects compared with other biorational or synthetic pesticides that are known to have quick knock down action. Furthermore, the extract application efficiency could be enhanced by incorporating mineral oils that improve plant coverage and penetration of chemicals into leaf surfaces (Bográn et al. 2006); knowing that *Melia* on tomato plants (Jazzar and Abou-Fakhr Hammad extracts were found to be enhanced by Tween-20 when applied against *B. tabaci*^2003^).

The compatibility of any potential and emerging pesticide with natural enemies is a basic component in building up Integrated Pest Management (IPM) programs. Villanueva-Jimenez and Hoy (1998) studied the relative toxicity of certain biorational pesticides to the CLM and its parasitoid *Ageniaspis citricola* using several bioassay methods. They classified azadirachtin (Neemix®) + oil, and oil alone (FC 435–66) as IPM-compatible insecticides, while azadirachtin (Align®) + oil and neem oil (Neemgard®) were ranked as semi-compatible insecticides, but avermectin (Agri-Mek®) + oil and imidacloprid (Provado®) when applied as sprays were considered as IPM-incompatible insecticides. The current results suggest that the *Melia* extract lends itself as a potential plant derived insecticide and merits further investigation in order to classify it as IPM compatible and use it with other control methods. The incorporation of these plant extracts might enhance the effect of natural enemies of the CLM under IPM programs; knowing that several predators and parasitoids (Karamaouna et al. 2010; Kalaitzaki et al. 2011; Tsagkarakis et al. 2013) were found to be important in reducing peak populations of the CLM, but apparently may need to be used in large numbers to provide good control of the pest (Knapp et al. 1995; Hoy and Nguyen 2006; Heppner and Fasulo 2010). Limited or no information is available not only on the natural enemies of the CLM in Lebanon, but also on the effect of botanical extracts on these beneficials. *Melia* extracts, however, have been tested against different natural enemy species. Percent parasitism of *Diglyphus isaea* (Walker) (Hymenoptera: Eulophidae), known to lower the populations of the leafminer *L. sativae* ( fruit extracts (Abou-Fakhr Hammad and McAuslane Diptera: Agromyzidae) was not affected by *Melia*^2010^). The same extracts were also found to have an additive effect on the whitefly *Bemisia argentifolii* Bellows & Perring nymphal mortality when combined with the parasitoid *Eretmocerus rui* Zolnerowich and Rose (Abou-Fakhr Hammad and McAuslane 2006). Furthermore, Melia extracts were found to be harmless to the predator, *Camptotylus reuteri* (Jakovlev), of *B. tabaci* as these extracts did not cause any mortality of the predator (Jazzar and Abou-Fakhr Hammad 2003). Thus, *Melia* extracts deserve further investigations taking into consideration the locally available CLM natural enemies in order to be used in IPM programs.

In conclusion, this work showed that *M. azedarach* extracts clearly had adverse effects on the *P. citrella* by decreasing the number of live CLM larvae (L1 and L2 & L3). These extracts are promising to be used in sustainable agriculture approaches against the CLM and deserve further investigations to fully elucidate their potential use alone and with biological control agents of this pest.
